# Role of CD44-Positive Extracellular Vesicles Derived from Highly Metastatic Mouse Mammary Carcinoma Cells in Pre-Metastatic Niche Formation

**DOI:** 10.3390/ijms25179742

**Published:** 2024-09-09

**Authors:** Ayana Ikari, Yuko Ito, Kohei Taniguchi, Masa-Aki Shibata, Kosei Kimura, Mitsuhiko Iwamoto, Sang-Woong Lee

**Affiliations:** 1 Department of General and Gastroenterological Surgery, Faculty of Medicine, Osaka Medical and Pharmaceutical University, 2-7 Daigaku-machi, Takatsuki 569-8686, Osaka, Japan; ayana.ikari@ompu.ac.jp (A.I.); kosei.kimura@ompu.ac.jp (K.K.); mitsuhiko.iwamoto@ompu.ac.jp (M.I.); sang-woong.lee@ompu.ac.jp (S.-W.L.); 2 Translational Research Program, Osaka Medical and Pharmaceutical University, 2-7 Daigaku-machi, Takatsuki 569-8686, Osaka, Japan; 3 Department of Anatomy & Cell Biology, Division of Life Sciences, Faculty of Medicine, Osaka Medical and Pharmaceutical University, 2-7 Daigaku-machi, Takatsuki 569-8686, Osaka, Japan; masaaki.shibata@ompu.ac.jp

**Keywords:** mouse mammary carcinoma cells, pre-metastatic niche, CD44-positive extracellular vesicles, CD44-CD44 homophilic interaction, hyaluronan, sentinel lymph node, vascular endothelial cell growth factor A (VEGF-A), vascular endothelial cell growth factor C (VEGF-C)

## Abstract

Malignant breast cancers pose a notable challenge when it comes to treatment options. Recently, research has implicated extracellular vesicles (EVs) secreted by cancer cells in the formation of a pre-metastatic niche. Small clumps of CD44-positive breast cancer cells are efficiently transferred through CD44–CD44 protein homophilic interaction. This study aims to examine the function of CD44-positive EVs in pre-metastatic niche formation in vitro and to suggest a more efficacious EV formulation. We used mouse mammary carcinoma cells, BJMC3879 Luc2 (Luc2 cells) as the source of CD44-positive EVs and mouse endothelial cells (UV2 cells) as the recipient cells in the niche. Luc2 cells exhibited an enhanced secretion of EVs expressing CD44 and endothelial growth factors (VEGF-A, -C) under 20% O_2_ (representative of the early stage of tumorigenesis) compared to its expression under 1% O_2_ (in solid tumor), indicating that pre-metastatic niche formation occurs in the early stage. Furthermore, UV2 endothelial cells expressing CD44 demonstrated a high level of engulfment of EVs that had been supplemented with hyaluronan, and the proliferation of UV2 cells occurred following the engulfment of EVs. These results suggest that anti-VEGF-A and -C encapsulated, CD44-expressing, and hyaluronan-coated EVs are more effective for tumor metastasis.

## 1. Introduction

Malignant breast cancers, such as triple-negative breast cancer (TNBC), that lack the estrogen receptor (ER), progesterone receptor (PR), and human epidermal growth factor receptor 2 (HER-2) as therapeutic targets exhibit a high proliferation capacity, a high metastatic potential, a high recurrence rate, and a poor prognosis. As a result, they have limited treatment options and are challenging to treat [1,2]. In recent years, there has been a growing interest in the properties of EVs and their role in metastasis. This has led to the proposal of anti-cancer agents based on their properties. In this study, we investigated the role of CD44-positive extracellular vesicles secreted by highly metastatic breast cancer cells (for, e.g., mouse mammary carcinoma cells using Luc2) in pre-metastatic niche formation. We sought to identify extracellular vesicles that could serve as more effective anticancer agents.

CD44 is a widely expressed cell-surface transmembrane glycoprotein composed of standard (CD44s) and variant (CD44v) forms. It is well-known that CD44 is a cancer stem cell marker and epithelial–mesenchymal transition factor in breast and other cancers [3,4,5,6]. Recent studies indicate that CD44 homophilic interactions (CD44–CD44) are essential for the collective migration and cohesive shedding metastasis of circulating tumor cells [7] and tumor-derived EVs [8,9]. Recent studies have established the relationship between CD44-positive exosomes and cancer progression. For example, CD44v6-positive exosomes secreted by pancreatic and colorectal cancer cells promote cancer invasion [10], CD44v8-10 mRNA in patient serum exosomes can be a diagnostic marker for docetaxel-resistant prostate cancer [11], CD44 in exosomes secreted by gastric cancer cells promotes lymph node metastasis [12], CD44 in exosomes secreted by highly metastatic ovarian cancer cells promotes the invasion of low metastatic ovarian cancer [13], and CD44 in the exosome secreted by breast cancer cells treated with doxorubicin CD44 promotes chemotherapy resistance [14]. Furthermore, anticancer drug-delivery systems using exosomes that target or utilize CD44 have been proposed [15,16]. CD44 is a receptor that internalizes hyaluronan (HA). HA is a glycosaminoglycan ubiquitously expressed in the mammalian extracellular matrix. Its primary function is to support cell adhesion and migration. It is also an essential mediator of cell migration during embryo morphogenesis, healing, and tumor metastasis in adults [17,18]. The CD44, lymphatic vessel endothelial HA receptor (LYVE-1), HA receptor for endocytosis (HARE), and receptor for HA-mediated motility (RHAMM) have been identified to bind to HA. Hyaluronidase in tissues rapidly metabolizes HA, allowing lower-molecular-weight hyaluronate to enter the lymph or bloodstream [19,20]. However, the function of CD44-positive extracellular vesicles in cancer pathogenesis, especially in pre-metastatic niche formation, remains uncertain. To address this, in vitro experiments were conducted using Luc2 cells (highly metastatic mouse mammary carcinoma cells) as donor cells of CD44-positive extracellular vesicles and UV2 cells (mouse hemangioendothelioma-like endothelial cells) as recipient cells, and optimal extracellular vesicle formulations were constructed. We used electron microscopy to investigate whether extracellular vesicles secreted by Luc2 cells formed clusters through CD44–CD44 homophilic protein interaction and whether hyaluronic acid promoted cluster formation. In addition, we will determine whether the HA receptor expressed by UV2 endothelial cells facilitates the uptake of extracellular vesicles secreted by Luc2 cells.

The most common organ metastases of breast cancer are hematogenous metastases to the lungs, liver, bone, and brain, depending on the subtype. Lymphatic metastasis to sentinel and axillary lymph nodes in breast cancer is common, and the lymph nodes are well-known as the first organ to metastasize. In 1889, Paget proposed the seed and soil hypothesis to explain nonrandom metastasis [21]. Filder et al. provided the first experimental evidence of organotypic metastasis [22]. Recent studies demonstrate that breast cancer subtypes, the host organ microenvironment, and cancer cell–organ interactions regulate organotypic metastasis [23,24]. Additionally, tumors establish a pre-metastatic niche before tumor cells reach the metastatic site. In pre-metastatic niches in sentinel nodes, the endothelial growth factor is expressed more strongly, and the lymphatic vessel density increases due to the formation of lymphatic vessels, remodeling of the extracellular matrix, and the influx of immunosuppressive cells [25,26,27]. To ascertain the point at which the pre-metastatic niche is formed, Luc2 cells were cultured under two distinct oxygen concentrations: 20% (representative of the early stages of tumor formation) and 1% (representative of the solid tumor stage). This allows for determining the point at which extracellular vesicles are secreted, facilitating the formation of the pre-metastatic niche.

Extracellular vesicles (EVs) are membrane-enclosed vesicles with a diameter of approximately 100 nm. Almost all cells in the body secrete EVs containing a wide variety of substances such as RNA, DNA, and lipids that are delivered to recipient cells, inducing various metabolic changes in them [28,29,30,31,32]. Recent studies suggest that tumor-derived exosomes are critical for organotropic metastasis. Tumor-derived EVs actively remodel distal organs to support pre-metastatic seeding by educating cells of metastatic sites [33]. They deliver seeding factors (proteins, metabolites, lipids, RNA, miRNA, and DNA) to turn distant microenvironments into favorable and fertile soil for tumor growth [24,34,35]. Breast cancer exosomes contribute to pre-metastatic niche-formation in bone [36] and lymph nodes [28,37]. For extracellular vesicles to induce metabolic or functional alterations in the target cell, they must first arrive at the target cell site and then undergo contact and fusion with the cell membrane of the target cell, resulting in the uptake of the extracellular vesicles by the target cell [38]. Endothelial cells (mouse endothelial cells; UV2 cells) were examined in propagation experiments to determine whether they could be the target cells of extracellular vesicles secreted by Luc2 cells expressing CD44.

The vascular endothelial growth factor (VEGF) family is crucial for niche formation during pathology and lymphangiogenesis. The VEGF family includes VEGF-A, VEGF-B, VEGF-C, VEGF-D, and placental growth factor (PIGF). Recent studies show that cancer cells (including breast cancer cells) secrete VEGF-A and VEGF-C. VEGF-A has four isoforms (VEGF-A-165, -121, -189, and -206), and VEGF-C undergoes multistep processing by proprotein convertase in producing cells to form a mature homodimer of 21 kDa. Both the mature and immature forms of VEGF-C are secreted [39]. According to the principles of neovascularization, VEGF-A binds to VEGFR-1 and VEGFR-2 to induce angiogenesis, and VEGF-C binds to VEGFR-3 to induce lymphangiogenesis [40,41,42]. Vascular endothelial cells express VEGFR-1, and VEGFR-2 is predominantly expressed in the activated endothelial cells. In contrast, the lymphatic endothelial cells express VEGFR-3 and VEGFR-2. VEGFR-3 binds to immature and mature VEGF-C, whereas VEGFR-2 only binds to mature VEGF-C [43]. In this study, we investigated whether vascular endothelial growth factor-A (VEGF-A) and -C, encapsulated by extracellular vesicles secreted by Luc2 cells, are taken up by UV2 endothelial cells and induce endothelial cell proliferation.

This study aims to elucidate the role of CD44-positive extracellular vesicles secreted by highly metastatic mouse mammary carcinoma cells (Luc2 cells) containing VEGF-A and VEGF-C in pre-metastatic niche formation. To ensure the highest level of accuracy, we carefully used an in vitro tumorigenesis model. In this model, the intra-tumor environment during pre-metastatic niche formation was assumed to have an average oxygen concentration (20% O_2_). The intra-tumor environment of solid tumors with advanced tumorigenesis was considered to have a hypoxic concentration (1% O_2_), and which environment better facilitated extracellular vesicle formation for niche formation was determined by the volume of VEGF-A and VEGF-C in the extracellular vesicles. In addition, we used UV2 endothelial cells as an example of vascular and lymphatic endothelial cells that form niches in lymph nodes to validate our methodology. Moreover, we put forward a new biomarker for non-invasive examination and therapeutic strategies using CD44-EVs.

## 2. Results

### 2.1. Determination of the Intratumor Microenvironment Suitable for Extracellular Vesicle Secretion and Formation of a Pre-Metastatic Niche

The objective of the first experiment was to ascertain whether Luc2 cells respond to hypoxia. The expression of HIF1-α, a typical hypoxia-inducible factor, was examined before the experiment (Supplemental Figure S1). The expression levels of the factors (CD44, VEGFs) required for pre-metastatic niche formation are elevated in extracellular vesicles secreted by Luc2 cells, under normoxia. It was postulated that pre-metastatic niche formation occurs in the early stages of tumorigenesis under normoxia, while hypoxia replicates the intratumoral microenvironment during solid tumorigenesis. According to the MTT assay conducted in this study, the proliferation rate of Luc2 cells was significantly higher under hypoxia than under normoxia (Supplemental Figure S1D). The western blot expression of HIF1-alpha, a representative hypoxia-inducible factor, showed that the percentage of activated hypoxia-inducible factor-alpha-positive cells localized to the nucleus by fluorescent immunostaining was more significant under hypoxia, but not significantly different between the two environments (Supplemental Figure S1A–C). Western blot analysis of the expression of another hypoxia-inducible factor, the MAPK-specific phosphatase dual-specificity phosphatase-2 (DUSP2), showed that its expression was decreased in hypoxia (Supplemental Figure S1E).

### 2.2. EVs Purified under Normoxic Conditions Were Advantageous for Pre-Metastatic Niche Formation

**Figure 1:**
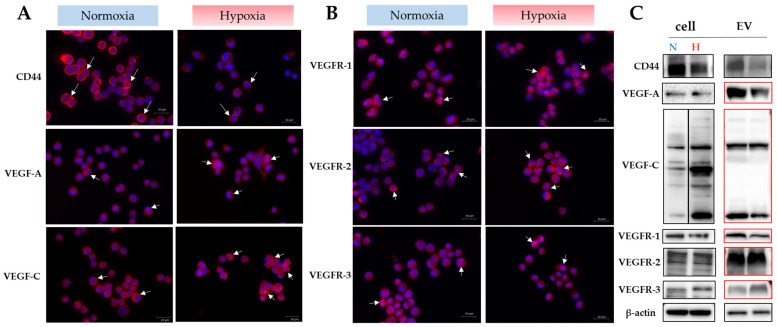
Expression of CD44, VEGF-A, and VEGF-C in Luc2 cells and EVs under normoxic and hypoxic conditions. (**A**,**B**) Immunofluorescent staining, with positive cells indicated by white arrows. Red fluorescence: positive area, blue fluorescence: DAPI (cell nucleus). (**C**) Western blotting. CD44, VEGF-A, and VEGF-C in Luc2 EVs under normoxic conditions showed more robust expression than in hypoxic conditions. The red box represents the results from western blot analysis using 20 μg of EVs. Scale bar = 20 μm.

Luc2 cells and EVs secreted by these cells express CD44, VEGF-A, VEGF-C, and receptors of VEGFs. The expression levels of CD44, VEGF-A, and VEGF-C in the EVs were higher under normoxia than hypoxia. However, their receptor expression in the EVs differed: VEGFR-1 was more strongly expressed under normoxia, VEGFR-3 was more strongly expressed under hypoxia, and the VEGFR-2 expression was equivalent under normoxia and hypoxia (Figure 1A,B). Notably, these receptor proteins could not be detected with the same amount of protein found for the cells. The doubling of the amount of cellular proteins has allowed for the identification of these receptor proteins (highlighted in red in western blot, Figure 1C). Therefore, extracellular vesicles obtained under normoxic conditions were utilized in subsequent experiments.

### 2.3. EVs Secreted by Luc2 Cells Meet the Exosome Requirements

**Figure 2:**
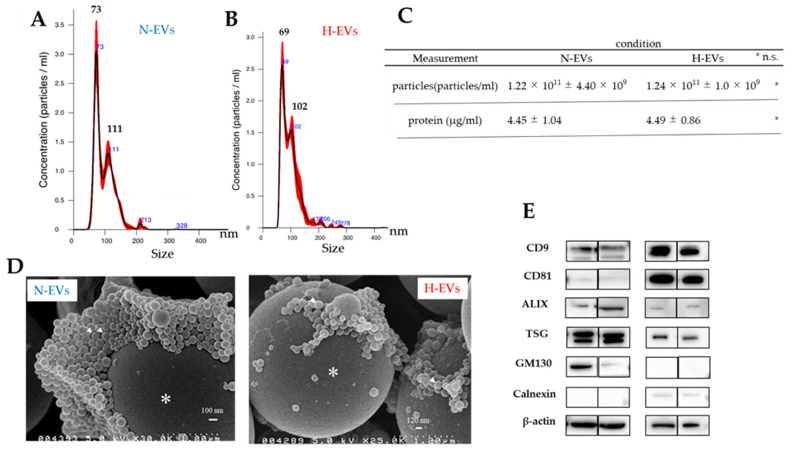
Our study meticulously employed precise methods to analyze the pathological features of Luc2-EVs. (**A**,**B**) Nanoparticle tracking analysis (NTA) of normoxia-EVs (N-EVs) (**A**) and hypoxia-EVs (H-EVs) (**B**). The black numbers indicate the peak of diameter of EVs. (**C**) NTA size assessment of N-EVs and H-EVs. NTA analysis showed that the mean diameter of N-EVs was 97.7 ± 0.6 nm, and for H-EVs, it was 97.4 ± 0.5 nm. The mode frequencies were 72.1 ± 0.7 nm and 67.6 ± 2.1 nm, respectively. (**D**) Scanning electron microscopy photos of N-EVs and H-EVs. White arrows indicated EVs. * indicated 3 μm-polyethylene beads coated with poly-L-lysine. Western blot analysis of EVs marker-related protein (**E**). N: normoxic condition, H: hypoxic condition.

There was no significant difference in the average diameters of the normoxic and hypoxic EVs. The nanoparticle tracking analysis (NTA) of untreated extracellular vesicles showed that the mean diameter of the extracellular vesicles was 97.7 ± 0.6 nm under normoxia and 97.4 ± 0.5 nm under hypoxia. The D10, D50, and D90 were 65.8 ± 1.1 nm, 88.3 ± 1.2 nm, and 138.4 ± nm, respectively, for extracellular vesicles under the average oxygen concentration, and 64.5 ± 1.0 nm, 90.5 ± 1.0 nm, and 137.4 ± 3.2 nm, respectively, under hypoxia. The number or concentration of EVs secreted by the Luc2 cells was 1.22 × 10^11^ ± 4.40 × 10^9^ particles/mL under normoxia and 1.24 × 10^11^ ± 1.0 × 10^9^ particles/mL under hypoxia, as determined by NTA analysis (Figure 2A,B). The protein levels were 4.45 ± 1.04 μg/mL under normoxia and 4.49 ± 0.86 μg/mL under hypoxia. Statistical processing by Student’s *t*-test showed no significant differences in both the particle number and protein levels (Figure 2C). Scanning electron microscopic (SEM) observation demonstrated particle images for EVs (white arrows in Figure 2D). Extracellular vesicles derived from Luc2 cells expressed the marker tetraspanins CD9 and CD81 (the usual markers for EVs and evidence of endosome origin of EVs), ALIX and TSG101 (proteins for marking of intraluminal vesicles in the multivesicular body), and GM13 and Calnexin (no expression means evidence of endosome origin), which is consistent with the MISEV guideline [28] (Figure 2E).

### 2.4. Role of CD44 on Extracellular Vesicles Derived from Luc2 Cells

#### 2.4.1. CD44 on the Luc2-EV Forms Aggregates among Luc2-EVs by Homophilic Protein Interaction (CD44-CD44)

**Figure 3:**
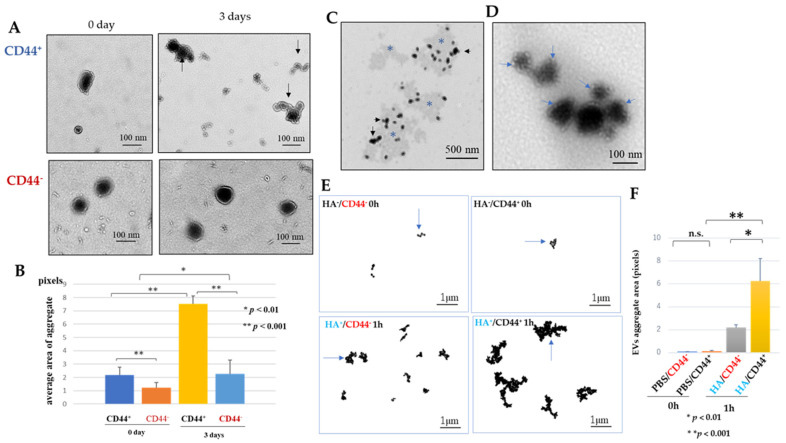
Luc2-EVs expressing CD44 form small aggregates via CD44 and HA (hyaluronan). (**A**) Analysis of CD44 on Luc2-EVs by transmission electron microscopy (TEM) for homophilic interaction between CD44 on Luc2-EVs. The top row shows EVs incubated in PBS (CD44^+^-EVs) for 0 and 3 days (black arrows indicate EV aggregates), and the bottom row shows EVs in PBS after treatment with a neutral anti-CD44 antibody (CD44^−^-EVs). (**B**) The bottom graph shows statistical analysis between CD44^+^-EVs and CD44^−^-EVs for forming EV aggregates. The average aggregate area was measured per pixel and displayed as pixels. * *p* < 0.01. ** *p* < 0.001. (**C**) TEM image of Luc2-EVs incubated in 0.05% HA solution for 7 days. Single or aggregated EVs (black arrowheads) were on the HA (high electron-density materials: blue asterisks). (**D**) TEM of hyaluronan-binding protein (HABP) in Luc2-EVs. Twenty nm gold (blue arrowheads) on the EVs demonstrated HA conjugated on the Luc2-EVs. (**E**) Analysis of the effect of HA on the aggregate, EVs were treated with or without HA (HA^+^, HA^−^) and anti-CD44 neutral antibody (CD44^+^, CD44^−^). The transmission electron micrograph depicts a clump of extracellular vesicles (blue arrows) observed in a single field of view at a magnification of 5000×. The area of the clump of extracellular vesicles observed in the field of view was subsequently measured using the ImageJ program 1.54j, a software program designed for image analysis. The surface area of clumps comprising two or more extracellular vesicles was quantified. (**F**) Statistical analysis of results are shown in (**E**). * *p* < 0.01. ** *p* < 0.001.

The homophilic protein interaction between CD44 and CD44 on Luc2-EVs was demonstrated in EVs incubated with phosphate-buffered saline (PBS) (Figure 3A). It was significantly prevented by adding a neutral anti-CD44 antibody (Figure 3B). Luc2-EVs were attached to the accumulated HA (Figure 3C). As the immunogold (conjugated with hyaluronan-binding protein) analysis shows (Figure 3D), Luc2-EVs were attached to the HA.

#### 2.4.2. Hyaluronan (Ligand for CD44) Contributes to Forming Small Clusters of Luc2-EVs

The size of the small mass area created when CD44 is removed from EVs with anti-CD44 neutralizing antibody and immersed in PBS (HA^−^/CD44^−^ 0 h) is not significantly different from the size of the small mass area created when CD44 is not removed and immersed in PBS (HA^−^/CD44^+^ 0 h) (Figure 3E,F). However, when the cells were immersed in hyaluronan solution for one hour at 37 °C without CD44 (HA^+^/CD44^−^ 1 h) or in hyaluronan solution for one hour at 37 °C with CD44 intact (HA^+^/CD44^+^ 1 h), the latter exhibited a significantly greater mass area (Figure 3E,F). This result may be attributable to the combined action of CD44 and HA.

### 2.5. Were Luc2-EVs Internalized by Cells of the Endothelial Niche Cells?

**Figure 4:**
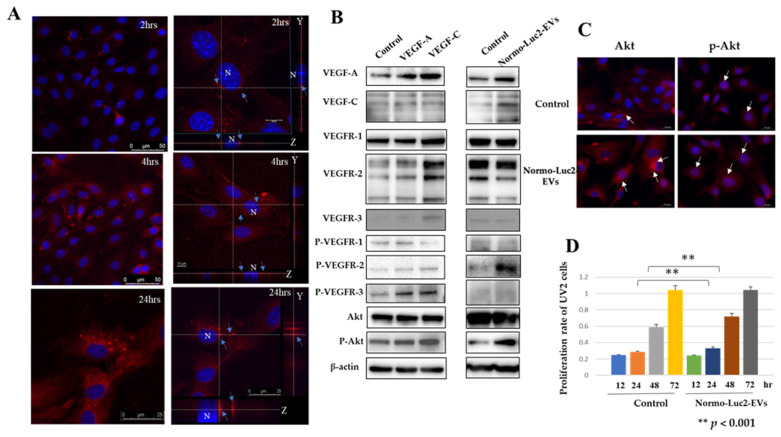
UV2 endothelial cells take up Luc2-EVs and show cell proliferation via vascular endothelial growth factors and their receptor axis. (**A**) Fluorescence images observed by confocal scanning laser microscopy. Red fluorescence: Luc2-EVs stained with ExoSparkler Red. Blue fluorescence: UV2 cell nucleus stained with DAPI. Right column: z-section of UV2 cell containing red Luc2-EVs. Blue arrows indicated X- or Y-sections of stained Luc2-EVs in UV2 cytoplasm. Scale bars: 2 and 4 h; left; 50 μm, right; 10 μm. (**B**) Western blot analysis. Left column: Expression of VEGF-A, VEGF-C, VEGFR-1, -2, -3 phosphorylated Akt, and Akt on adding recombinant VEGF-A and VEGF-C to UV2 endothelial cells. Right column: expression of VEGF-A, VEGF-C, VEGFR-1, -2, and -3, phosphorylated Akt, and Akt upon the addition of Luc2-EVs purified under normoxia (Normo-Luc2-EVs). (**C**) Immunofluorescent analysis. Akt and phosphorylated Akt were expressed by adding Normo-EVs. White arrows indicate a high expression level of Akt or phosphorylated Akt (red fluorescent). Nuclei were counter-stained by DAPI. Scale bar = 20 μm. (**D**) Our MTT assay shows the cell-proliferation rate of UV2 cells after adding Normo-Luc2-EVs. Results were obtained after 24 and 48 h.

We used mouse UV2 endothelial cells (UV2 cells) derived from ultraviolet-induced hemangioendothelioma-like tumors as recipient cells for Luc2-EVs uptake experiments. The UV2 endothelial cells showed CD44 and hyaluronan receptor of endocytosis (HARE) expression on the cell membrane and intracellular localization (Supplemental Figure S2A). They also exhibited the cytoplasmic expression of VEGF-A, VEGF-C, and their receptors (Supplemental Figure S2B). Therefore, given that UV2 endothelial cells express VEGF-A, -C, and their receptors, it is imperative to consider this factor when interpreting the subsequent experimental outcomes on extracellular vesicles. Scanning in the x- and y-axes by laser microscopy showed red vesicles in the cytoplasm around the nucleus, confirming that the EVs of Luc2 were incorporated into the UV2 cells (Figure 4A).

#### 2.5.1. VEGFs Internalized by Luc2 Extracellular Vesicles May Act on UV2 Endothelial Cell Metabolism

To determine whether the respective endothelial growth factor receptors (VEGFR-1, VEGFR-2, and VEGFR-3) expressed on the UV2 cells were responsive to each endothelial growth factor, recombinant VEGF-A and VEGF-C proteins were added to UV2 cells and cultured under normoxic conditions. The expression of each phosphorylated receptor was more potent than that of the control (PBS) (Figure 4B, left column). The phosphorylated Akt expression was more robust than that of the control PBS, especially with the addition of VEGF-C (Figure 4B, left column). When we placed the EVs collected under normoxia or hypoxia conditions on UV2 cells, the expression of VEGF-A, VEGF-C, VEGFR-1, -2, phosphorylated VEGFR-2, and phosphorylated Akt slightly increased in the UV2 cells compared to the PBS control (Figure 4B, right column). The propagation of Luc2-EVs resulted in Akt translocation to the nucleus according to fluorescence immunostaining. When Luc2-EVs collected under normoxic and hypoxic culture conditions were propagated in UV2 endothelial cells, Akt expression was observed in the cytoplasm; however, phospho-Akt was also present in the nucleus. In contrast, the phospho-Akt expression in the nuclei of PBS-supplemented controls showed a punctate pattern, and the Luc2-EV-uptake groups clumped together as thick strings (Figure 4C). The MTT assays (cell-proliferation assay) showed that the amount of UV2 endothelial cells increased after 24 and 48 h when EVs were added (Figure 4D).

#### 2.5.2. Some Extracellular Vesicles Are Readily Internalized by CD44-Positive UV2 Endothelial Cells, Hyaluronan-Coated or Non-Coated EVs

**Figure 5:**
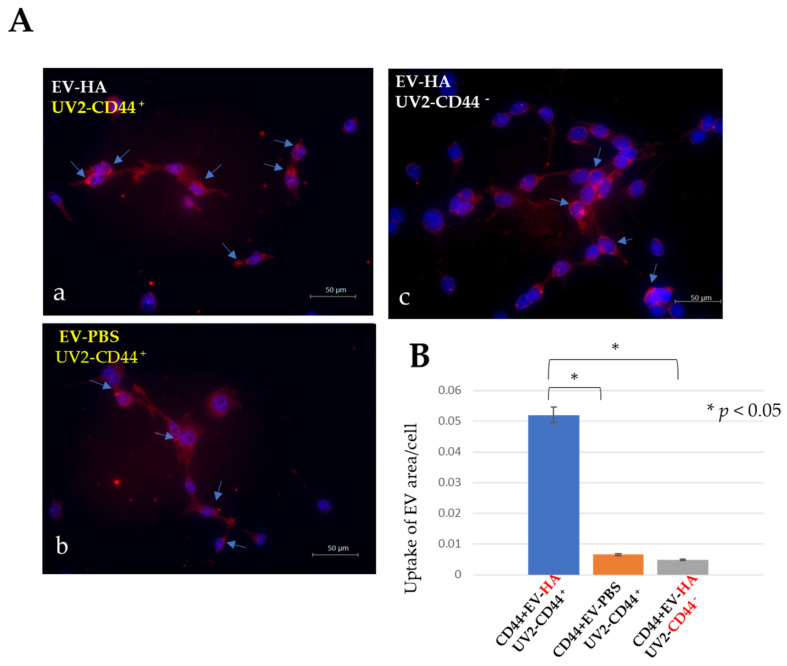
Assessing the effect of an HA coat on the uptake of Luc2-EVs by UV2 endothelial cells. (**A**) Immunofluorescence micrographs of internalized EVs stained with ExoSparkler red (blue arrows). The propagation conditions were EVs coated with HA in (**a**), uncoated (PBS) EVs in (**b**), and EVs coated with HA and CD44-negative UV2 in (**c**). Blue arrows point to internalized EVs. (**B**) Statistical analysis of internalized EVs. *: *p* < 0.05. Scale bar = 50 μm.

The HA treatment of EVs significantly increased their uptake by endothelial cells (Figure 5(Aa)). The hyaluronic acid-treated extracellular vesicles showed a considerably lower uptake rate when the endothelial cells were treated with CD44-neutralizing antibodies (Figure 5(Ac)). Similarly, untreated UV2 endothelial cells showed a low uptake of untreated EVs (Figure 5(Ab)). A statistical analysis conducted using ImageJ revealed that the UV2 cells exhibited a high degree of uptake of hyaluronan-coated Luc2-EVs (Figure 5B).

## 3. Discussion

We investigated the role of CD44-positive EVs secreted by highly metastatic mouse mammary carcinoma cells in forming a premetastatic niche. CD44-positive EVs formed small clumps through CD44–CD44 homophilic interactions. They also adhered to hyaluronan. Endothelial cells expressing the hyaluronan receptors CD44 and HARE took up many CD44-positive EVs when coated with hyaluronan. Endothelial cells take up EVs and phosphorylate VEGF-A or VEGF-C receptors, increasing their proliferation.

### 3.1. Luc2-EVs Collected Met the Criteria for Extracellular Vesicles Defined in the Minimal Information for the Studies of Extracellular Vesicles (MISEV) Guidelines

The EVs collected from the culture supernatant of Luc2 cells met the definition of EVs proposed at the ISEV meeting [28,29,30]. Based on the ISEV meetings, the EVs collected by ultracentrifugation were negative for GM130 and calnexin proteins according to western blot analysis, indicating that they were not intracellular vesicles. Positive ALIX and TSG101 proteins and MVB detected by electron microscopy also showed an endosomal origin. The EVs were either smaller than 100–120 nm or larger, based on nanoparticle tracking analysis (NTA), scanning electron microscopy (SEM), and TEM [44,45,46]. The tetraspanins CD9, CD63, and CD81 are present on the membranes of EVs. Western blot analysis showed that the Luc2 mouse mammary carcinoma cells expressed CD9 and CD81 but not CD63. Since MISEV 2014 [28], when the ISEV proposed a definition of EVs, several subtypes have been reported, including tetraspins [47,48,49]. Currently, MISEV 2018 and MISEV 2023 do not propose specific molecular markers for characterizing each EV subtype. Based on the MISEV proposal and these studies, the CD9 and CD81 of EVs secreted by Luc2 cells may suggest several subtypes of this EVs.

### 3.2. Extracellular Vesicles, Normally Secreted under Oxygenated Conditions, Facilitated Pre-Metastatic Niche Formation

In this study, we used an in vitro model to reproduce the time course of tumorigenesis. Normoxic conditions represent the early stage of tumorigenesis, and hypoxic conditions represent the late stage of solid tumors. We investigated whether Luc2 cells respond to hypoxia. There was no significant difference in the expression of HIF1-alpha in Luc2 cells under normoxia and hypoxia, but the expression of DUSP2, another factor expressed by hypoxia, was decreased under hypoxia. Since the downregulation of DUSP2 induces VEGF-C expression [50,51], which was the case in our experiments, we hypothesized that Luc2 cells are hypoxia-responsive. The lack of significant differences in the EVs secretion (number of particles and protein concentration) under normoxia and hypoxia may be due to quantitative differences in the types of proteins encapsulated in the EVs, as described below. For pre-metastatic niche formation, EVs produced under normoxia are advantageous. The expression of VEGF-A, VEGF-C, and CD44 in EVs secreted by Luc2 cells was enhanced under normoxia compared to that under hypoxia, indicating that EVs contain many factors necessary for metastasis at the early stage of tumorigenesis. This suggested that EVs contain factors critical for metastasis at the early stage of tumorigenesis and strongly indicated that EVs are involved in pre-metastatic niche formation. In vivo experimentation revealed that the transplantation of Luc2 mammary carcinoma cells into female mice resulted in notable lymphatic sinus hyperplasia within the sentinel lymph nodes despite the absence of metastatic cancer cells in these nodes [52]. The presence of their receptors (VEGFR-1, -2, and -3) in cells strongly indicates the encapsulation of VEGF-A and VEGF-C in Luc2-EVs [28,29]. The sentinel lymph node is the first metastatic organ involved in lymphangiogenesis, and extensive endothelial venule (HEV) remodeling is a critical event in the formation of the lymph node pre-metastatic niche. VEGF-A and VEGF-C are responsible for inducing lymphangiogenesis involved in sentinel lymph nodes owing to the VEGFR-2 and -3 expression on sinusoidal lymphatic endothelial cells and are also responsible for inducing pre-metastatic niche formation, including HEV remodeling [53,54]. Taken together, these reports suggest that Luc2-secreted EVs participate in pre-niche formation.

### 3.3. Luc2 Extracellular Vesicles Were Observed to Form CD44-Mediated Clumps, and Hyaluronan Was Found to Promote Clump Growth

The idea that circulating tumor cells (CTCs) shed cohesively from the tumor and migrate collectively rather than migrating individually is proposed in studies on clinical specimens from patients with breast cancer [7]. This aggregation, called the CD44–CD44 protein interaction, induces the activation of PAK2 (p21-activated kinase 2) and FAK (focal adhesion kinase). Additionally, CD44-mediated cell aggregation through CD44–CD44 homophilic protein interactions was reported in vitro in MDA-MB-231 breast cancer cells. This aggregation does not involve other adhesion molecules, and anti-CD44 neutralizing antibodies block this aggregate [7]. We hypothesized that CD44-positive EVs (as well as CD44-positive cells) form CD44-mediated EV aggregations. When we treated EVs with anti-CD44 neutralizing antibodies, the aggregation was significantly reduced compared to when the EVs were not treated. These results strongly suggest the occurrence of the CD44-mediated aggregation of EVs. However, after three days of treatment with CD44 neutralizing antibody, the aggregation area was significantly larger (*p* < 0.01). This increase may be owing to the persistence of the neutralizing ability of the anti-CD44 neutralizing antibody or the aggregation of the EVs due to the heterophilic interaction of CD44 and CD81 [55]. CD44-positive EVs internalized and transported VEGF and VEGF-C to endothelial cells in our experiments. Using siRNA-CD44-EV (siRNA against CD44), MCF-7 breast cancer cells secreted CD44-positive EVs elicited by doxorubicin and carried proteins that confer drug resistance to breast cancer cells [14]. This experiment supported our results by showing that CD44-positive EVs secreted by breast cancer cells are essential for intercellular communication. In the experiment, as evidenced by Figure 3E,F, we show that HA promotes EVs aggregation. The predominance of a larger aggregate area when immersed in HA for one hour (HA^−^/CD44^+^ vs. HA^+^/CD44^−^) may indicate that factors other than CD44 are at work despite excluding CD44. HA, a large mucopolysaccharide (103–104 kDa), is an important structural component found in the connective tissue, synovial fluid, and vitreous body [17]. The estimated tissue HA turnover rate was approximately 50% per day. Hyaluronan produced in peripheral tissues has a molecular weight of 1000 kDa or greater. It is degraded by tissue cells to intermediate-sized HA, most of which enter the lymphatic vessels (>80%). This substance then undergoes further degradation within the lymph nodes. HA, with a molecular weight of under 10 kDa, enters the bloodstream and is eventually metabolized and excreted by the liver, kidneys, and spleen without degradation in the lymph nodes. Hyaluronic acid in primary tumors may undergo similar metabolic processes. The lymphatic flow from the primary tumor to the sentinel node may be a pre-metastatic pathway for EVs that cannot move independently [8,19,20]. The fact that the EVs formed small clusters on the HA in this experiment supports the transport of CD44-positive-EVs by HA. Moreover, the report indicated that HA is not degraded in the lymph nodes but is absorbed into the bloodstream, suggesting that EVs may enter the bloodstream from the lymph nodes.

### 3.4. Hyaluronan Receptors (CD44, HARE, and LYVE-1) on Vascular Endothelial Cells Facilitate the Take-Up of EVs

In this study, CD44 and HARE were investigated as HA receptors mainly expressed in vascular endothelial cells in blood vessels [56]; however, we did not investigate the HA receptor expressed in lymphatic endothelial cells, LYVE-1. CD44 and LYVE-1 are membrane-expressed proteins with similar HA-binding domains that bind and internalize soluble and immobilized HA. LYVE-1 is expressed on the luminal and basolateral sides of the lymphatic endothelial cells, allowing HA to move from the tissue into the lymphatic lumen. Furthermore, LYVE-1 has no variants, whereas CD44 has several variants and CD44-positive capillaries are found in various cancers, including breast cancer. HARE is found in the sinusoidal endothelia of the lymph nodes, liver, and spleen and the vascular endothelia of the eye, brain, stomach, and heart. Unlike LYVE-1 and CD44, HAREs can bind to other glycosaminoglycans (GAGs), suggesting that the clearance of GAGs in the circulating blood may play a significant role in HARE formation. The central role of HARE is the clearance of GAGs from the circulating blood. Thus, all three contribute to HA incorporation into the endothelium, although the modes of HA uptake are different [19,56]. The endothelial cells in this study (UV2) expressed CD44, the same as tumor blood vessels [57], HARE, and internalized HA. Additionally, EVs bound to HA were more readily taken up by the endothelium than untreated EVs, and the uptake rate of HA-treated extracellular vesicles was reduced in the absence of an HA scavenger, supporting the existence of the HA-mediated uptake of EVs in blood and lymphatic vessels [58]. However, phagocytosis may also play a role in uptake (Figure 4A), wherein many UV2 endothelial cells engulf red fluorescent extracellular vesicles [54]. Furthermore, homophilic and heterophilic protein interactions between CD44 expressed by UV2 endothelial cells and CD44 and CD81 expressed by extracellular vesicles may contribute to their uptake [7,8,55].

### 3.5. UV2 Endothelial Cells Take Up Normoxic EVs and Proliferate

For EVs to effectively function in recipient cells, they must enter the cytoplasm of the recipient cell. There are two delivery routes for EVs. In the first, the EVs membrane fuses with the plasma membrane of the recipient cell, releasing its contents into the cell. In the second, the contents of EVs are taken up by endosomes or phagosomes and released into the cytoplasm by fusion of the endosome membrane or phagosome with those of the EVs [59,60,61,62]. In both cases, the reaction occurred in the cytoplasm of the receptor cells. In this experiment, the amount of phagosomes containing EVs stained with red fluorescent dye increased over time. These results suggested that phagosomes release their inclusions into the cytoplasm, causing various cytoplasmic reactions.

The propagation of Luc2-EVs collected under normoxic conditions into UV2 endothelial cells increased the expression of VEGF-A and VEGF-C in UV2 endothelial cells, confirming the phosphorylation of these receptors. The increase in phosphorylated VEGFR-2 expression was particularly significant. However, the phosphorylated VEGFR-1 and VEGFR-3 expression was minimal, even at the maximum amount of protein used at one time (20 μg) in western blots. VEGFR-2 is the second VEGF-C receptor predominantly expressed in activated blood vessels and used by VEGF-A. The VEGFR-2 system may have worked [40,43]. However, this may be because the amount of each factor encapsulated in the EVs was variable, due to the heterogeneity of EVs. This may also be owing to factors that suppress growth (such as miRNAs). Extracellular vesicles are known for their heterogeneity as they differ in size, content, and other characteristics. However, after cell uptake, they may undergo some degree of digestion. After EVs spread, the subcellular localization of phosphorylated Akt in the UV2 endothelial cells shifted from the cytoplasm to the nucleus. Furthermore, this nucleus localization differed from that of the controls. This suggested that phosphorylation is induced by factors present in EVs. The MTT assay confirmed the proliferation of UV2 after Luc2-EVs propagation.

### 3.6. Extracellular Vesicles Expressing CD44 May Serve as a Potential Therapeutic Agent for Breast Cancer

Anti-VEGF-A and anti-CD44v6 antibodies target angiogenesis in cancer tissue and are used for cancer treatment [63,64]. Molecular targeted therapies have attracted attention, with anti-HER2 agents, CDK4/6 inhibitors, PARP inhibitors, and mTOR inhibitors being used to treat breast cancer. These therapies have become useful, but the treatment options still require improvement [1,2]. Using nanomedicines with EVs in cancer therapy has recently been considered [65,66]. Several studies have investigated the use of EVs in breast cancer [67,68]. There are two main types of nanomedicines. One is an EV mimetic, a modified version of an EV derived from the human body [28,69]. The other is an artificially engineered EV, wherein the EV membrane is replaced by a mimetic liposome filled with therapeutic agents (such as VEGF-A-siRNA and various miRNAs) [70,71]. Based on the results of this study, we propose an EV mimetic membrane as a pre-metastatic niche inhibitor with HA and CD44 as surface modifications and the loading of therapeutic agents, and we suggest anti-VEGF-A and VEGF-C antibodies and miRNAs targeting VEGF-A and VEGF-C as agents for loading. Additionally, a signal is attached to the macrophages on the EV membrane surface, saying, “Do not eat me” (such as CD47).

### 3.7. Limitations

First, lymphatic endothelial cells were not used. Lymphatic endothelial cells express LIVE-1 as an HA receptor on the luminal and basolateral sides; therefore, we could not examine the effect of the expression on the basolateral side. Additionally, we could not replicate the effects of lymphatic flow on EVs assembly. Second, although we attempted to overcome the heterogeneity of individual EVs in the propagation experiments by increasing the volume of EVs added, we could not eliminate the effect of the individual heterogeneity of EVs on the results. This in vitro model does not show how EVs enter the lymphatic or blood circulation and reach the lymph nodes with HA. This is a crucial aspect that could be addressed in future experiments using an in vivo model, i.e., studying the migration of EVs with a marker indicating that they were secreted from the transplanted tumor. Such studies could significantly enhance our understanding of the metastatic process and the role of EVs in them.

**Figure 6:**
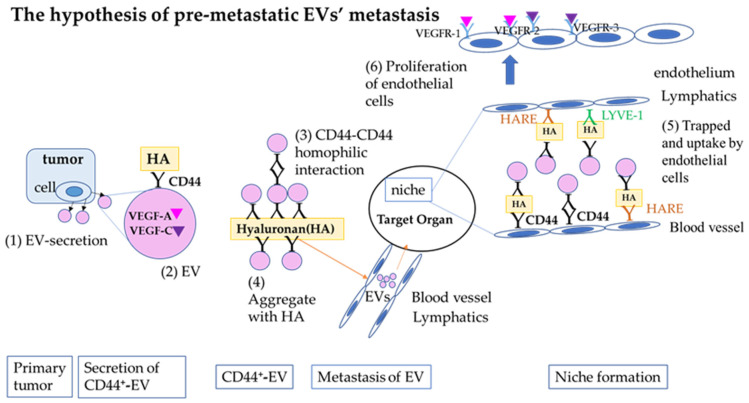
Hypothesis of pre-metastatic EVs’ metastasis. (1) EV secretion in the early stage of tumorigenesis. (2) EVs contained VEGF-A, VEGF-C, and VEGFR-1, -2, and -3. EVs expressed CD44, which is an HA receptor. (3) EVs aggregated by CD44–CD44 interaction. (4) The aggregates of EVs are attached to HA, and EVs enter the vasculature with hyaluronan, the beginning of pre-metastatic EVs’ metastasis. (5) In this organ, EVs are trapped by endothelial cells that express HA receptors. (6) Endothelial proliferation induced by VEGF-A, VEGF-C, and their receptors: the final stage of premetastatic niche formation.

In summary, highly metastatic mouse mammary carcinoma cells express the hyaluronan receptor CD44 and secrete CD44-positive EVs. These EVs are thought to play a role in effectively transporting VEGF-A and VEGF-C (necessary for pre-metastatic niche formation) by forming small aggregates and delivering them to target endothelial cells with hyaluronan (Figure 6). These CD44-positive EVs can serve as inhibitors of pre-metastatic niche formation by encapsulating niche-blocking drugs.

## 4. Materials and Methods

### 4.1. Cell Culture and Cell Preparation

We used the BJMC3879Luc2 mammary carcinoma cell line (Luc2 cells) as donor cells for supplying CD44-positive EVs and UV2 mouse angioendothelioma-like endothelial cells (RIKEN BRC, Tsukuba, Japan) for the recipient cells receiving EVs. The Luc2 cell line was generated from the BJMC3879 mammary carcinoma cell line, mL, derived from BALB/c mice by transfection of the luc2 gene and showed a high metastatic propensity to the lymph nodes and lungs [72]. Luc2 cells were cultured in RPMI-1640 medium (FUJIFILM Wako Pure Chemical Corporation, Osaka, Japan) supplemented with 10% fetal bovine serum. The UV2 cell line was derived from an ultraviolet-induced angioendothelioma-like tumor in an immunocompetent CB6 mouse (F1 from BALB/c × C57BL/6) and cultured in Dulbecco’s modified Eagle’s medium (DMEM; low glucose) (FUJIFILM Wako Pure Chemical Corporation) supplemented with 10% fetal bovine serum. Both cell lines were cultured under normoxia (20% O_2_) or hypoxia (1% O_2_) [73] conditions at 37 °C in a 5% CO_2_ incubator.

### 4.2. Extracellular Vesicle Isolation

BJMC3879Luc2 cells were cultured in RPMI-1640 medium supplemented with 10% fetal bovine serum without endogenous bovine extracellular vesicles (System Biosciences, LLC, Palo Alto, CA, USA) for 72 h. The culture medium was collected and centrifuged at 2500 rpm for 5 min. The resulting supernatant was filtered through a 0.45 μm pore filter to remove cellular debris and a 0.20 µm pore filter to isolate exosome-sized EVs. The flow-through fraction was ultra-centrifuged at 100,000× *g* for 4 h at 4 °C. The supernatant was carefully removed, and the resulting pellets were used for subsequent experiments. We named the EVs purified from Luc2 cells cultured under normoxic conditions normal EVs and hypoxia-EVs derived from Luc2 cells cultured under hypoxia.

### 4.3. Nano Tracking Analysis (NTA)

NTA measurements were performed using NanoSight NS300 (NanoSight, Ltd., Amesbury, Wiltshire, UK). The EV pellets suspended in 1 mL of PBS were analyzed using the NTA Version 3.4 Build 3.4.003-SA instrument. The following photographic conditions were used: frames processed (1498), frames per second (25), calibration (190 nm/pixel), and detection threshold (5 or 6).

### 4.4. Scanning Electron Microscopy (SEM)

Luc2 cell-EV pellets suspended in PBS were fixed in 1.25% glutaraldehyde (pH 7.4) for 15 min and then put on 3 μm polyethylene beads coated with poly-L-lysine. The specimens were rinsed with PBS, post-fixed in 1% osmium tetroxide (pH 7.4) for 20 min, and dehydrated using a graded ethanol series. After dehydration, the samples were coated with platinum-palladium and observed using SEM (Hitachi S-5000: Hitachi, Tokyo, Japan).

### 4.5. Western Blotting Analysis

Samples were lysed with chilled radioimmunoprecipitation assay (RIPA) buffer (Thermo Fisher Scientific Inc., Waltham, MA, USA) and 1% protease inhibitor cocktail (Sigma-Aldrich Co. LLC, St. Louis, MO, USA) and incubated for 20 min on ice. After centrifugation at 12,000 rpm for 20 min at 4 °C, the filtrates were measured with a DCTM protein assay kit (BIO-RAD, Laboratories, Inc., Hercules, CA, USA). Samples containing from 6 to 20 μg of protein were fractionated in 7.5–12.5% polyacrylamide gels (FUJIFILM Wako Pure Chemical Corporation) and transferred onto a polyvinylidene difluoride (PVDF) membrane (PerkinElmer Life Sciences, Inc., Waltham, MA, USA). After blocking nonspecific binding sites with 5% nonfat milk or phosphoBLOCKER blocking reagent (Cell Biolabs, Inc., San Diego, CA, USA) in PBS containing 0.1% Tween 20 (PBS-T) or PVDF blocking reagent for Can Get Signal (TOYOBO Co., Ltd., Osaka, Japan), the membrane was incubated overnight at 4 °C with the primary antibodies, which were diluted in Can Get Signal Immunoreaction Enhancer Solution (TOYOBO Co., Ltd.). The following primary antibodies were used: anti-CD81, GM130, calnexin (Santa Cruz Biotechnology, Inc., Dallas, TX, USA); CD9, ALIX, TSG (Abcam, Cambridge, UK); anti-VEGF-A (Oncogene research products, Cambridge, MA, USA, 1:50), vascular endothelial cell growth factor receptor 1 (VEGFR-1) (Abcam, PLC, 1:50), VEGF-C, VEGFR-2, VEGFR-3 (Santa Cruz Biotechnology, Inc., Dallas, TX, USA, 1:50), CD44 (Abcam, 1:50), serine/threonine kinase (Akt), phosphorylated Akt (Phospo-Akt) (Cell Signaling Technology, Inc., Danvers, MA, USA, 1:50), phospho-VEGFR-1 (Tyr1333, Merck SA, Darmstadt, Germany), -2 (Tyr1175, CST), -3 (Tyr1230, Affinity Biosciences, Cincinnati, OH, USA), and HARE (Bioss antibodies, Woburn, MA, USA, 1:50), with β-Actin used as internal control. The membranes were washed with PBS-T and incubated for 1 h with horseradish peroxidase-conjugated horse anti-mouse and anti-rabbit secondary antibodies (CST). The immunoblots were visualized using Luminata™ Forte Western HRP Substrate (Millipore Corporation, Billerica, MA, USA) by chemiluminescence. Tris-buffered saline contains 0.1% Tween 20 (TBS-T) and PhosphoBLOCKER™ Blocking Reagent (San Diego, CA, USA) for phosphorylated protein.

### 4.6. Single and Double-Labeling Immunofluorescent Study

Luc2 cells were exposed to normoxic (20% O_2_) or hypoxic (1% O_2_) conditions for 72 h. Following fixation with a 4% paraformaldehyde solution (PFA) (pH 7.4) (FUJIFILM Wako Pure Chemical Corporation) for 10 min, the cells were washed in PBS for 15 min. The samples were incubated on ice in 0.5% Triton X-100 for 5 min. The samples were exposed to serum-ready-to-use protein block (Agilent Technologies, Inc., Santa Clara, CA, USA) for 20 min. Samples were incubated with primary antibodies overnight at 4 °C in a Buffer solution of anti-VEGF-A (oncogene research products, Cambridge, MA, USA, 1:50), -VEGFR-1 (Abcam, Cambridge, UK, 1:50), -HIF-1-α, -VEGF-C, -VEGFR-2, -VEGFR-3, -DUSP2 (Santa Cruz Biotechnology, Inc., Dallas, TX, USA, 1:50), -CD44 (TONBO Biosciences, San Diego, CA, USA, 1:50), Akt, Phospho-Akt (Cell Signaling Technology, Inc., Danvers, MA, USA, 1:50) and hyaluronan receptor of endocytosis (HARE) (Bioss antibodies, Woburn, MA, USA, 1:50). For visualization of hyaluronan localization, avidin conjugated hyaluronan-binding proteins (Merck SA, 1:50) and streptavidin Alexa Fluor™ 488 conjugate (Thermo Fisher Scientific Inc.) were used. Samples were washed in PBS, followed by incubation with Alexa Fluor™ 568 goat anti-mouse, Alexa Fluor™ 568 goat anti-rabbit, and Alexa Fluor™ 594 goat anti-rat antibodies (Thermo Fisher Scientific Inc., Waltham, MA, USA, 1:250) at room temperature (RT) for 1 h and counterstained with 4′,6-diamidino-2-phenylindole (DAPI) (VECTOR Laboratories, Newark, NJ, USA) for 15 min. Images were captured using a BZ-x700 microscope (KEYENCE, Tokyo, Japan) and confocal laser scanning microscopy (CLSM, TCS SP8, Leica GmbH, Wetzlar, Germany).

### 4.7. Analyzing CD44’s Ability to Bind with HA and Homophilic Interactions (CD44-CD44) on Luc2-EVs and UV2 Endothelial Cells

Luc2-EVs (10 μg/mL protein) incubated in 0.05% HA (FUJIFILM Wako Pure Chemical Corporation) in PBS for seven days was dropped onto an excel support film coated mesh (Nisshin EM, Tokyo, Japan) and fixed with 1.25% glutaraldehyde for 10 min. After washing for labeling HA, the sample was exposed to avidin-HABP and then to 20 nm immuno-gold-conjugated streptavidin. After 3 min of Pb staining, the samples were observed using transmission electron microscopy (TEM) (H-7800 Hitachi, Japan). To study CD44–CD44 homophilic interactions, Luc2-EVs were incubated in 20 μg/mL anti-mouse CD44 affinity-purified rabbit polyclonal antibody for 1 h in a 5% CO_2_ incubator at 37 °C immediately after purification (R&D Systems, Minneapolis, MN, USA) and stored at 4 °C for 0 and 3 d. Luc2-EVs suspended in PBS were used as the controls. Five microliters of the samples were dropped onto the Excel support mesh, briefly stained with Pb, and observed under TEM. Induction of Luc2-EVs aggregates by homophilic interaction between CD44 on Luc2-EVs. The EV-aggregates in the 10 fields of TEM photos under 5000× magnification were captured using Adobe Photoshop software version 25.11 (San Jose, CA, USA), and the mean area of clumps of EV-aggregates were counted by pixel using ImageJ (NIH, Bethesda, MD, USA). Data were analyzed using StatMate III version 3.14 (ATMS, Chiba, Japan). Statistical significance was determined using a two-sided Student’s *t*-test. Values are presented as the mean ± standard deviation. A *p* value < 0.05 was considered statistically significant.

### 4.8. Detecting the Vascular Endothelial Growth Factors and HA Receptor and Their Receptor-Expression Profile of UV2 Cells

Western blotting and single-labeling immunofluorescence studies were used to detect VEGF-A and VEGF-C and their receptors (VEGFR-1, -2, -3). The expression of CD44, HARE, and HA receptors was detected by western blotting and single- and double-labeling immunofluorescence using the aforementioned methods. Before double-labeling the HA receptors and HABP, the cells were cultured in a medium containing 0.05% HA for 72 h.

### 4.9. Uptake of EVs by UV2 Cells

To confirm the cellular uptake capacity of UV2 cells for Luc2-EVs labeled with ExoSparkler Exosome Membrane Labelling Kit-Red (Dojindo Molecular Technologies, Inc., Rockville, MD, USA), 1–5 μg/mL (measured as protein) of Luc2-EVs was added to the culture medium and cultured with UV2 cells for 0, 2, 4, 24 h in normoxia. The samples were examined using a BZ-x700 (KEYENCE) and a confocal laser-scanning microscope (TCS SP8, Leica). Before the uptake experiments, we investigated the efficacy of VEGFs in UV2 cells using murine recombinant VEGF-A (Sigma) and VEGF-C (BioLegend, San Diego, CA, USA). UV2 cells (1 × 10^6^) were cultured overnight under normoxic conditions in a medium supplemented with 5.0–20 ng/mL VEGF-A or VEGF-C. Phosphate-buffered saline was added to the medium as a control. After incubation in normoxia, western blotting analysis was performed on protein samples for anti-VEGF-A, -VEGF-C, -phospho-VEGFR-1, -2, -3, -phosphor-Akt, and -Akt according to the aforementioned western blot analysis. In the luc2-EVs uptake experiment for UV2, we added 5–10 μg (measured as protein) normoxia-EVs or hypoxia-EVs to UV2 cell medium and incubated them in normoxia condition overnight. Phosphate-buffered saline was added to the medium as a control. After exposure to EVs, western blot analysis was performed on the protein samples using the antibodies mentioned above.

### 4.10. Effect of EVs Uptake on UV2 Cells

The impact of UV2 uptake by EVs harvested from Luc2 cells was also examined. EVs were disseminated into UV2 cells in the same manner as described above, and fluorescent immunohistochemical staining for Akt and phosphorylated Akt was performed. Cell-proliferation rates were examined using 3-(4,5-dimethylthiazol-2-yl)-2,5-diphenyltetrazolium bromide (MTT). The UV2 cells were seeded in 12-well plates at a density of 1 × 10^5^ cells per well and cultured in RPMI-1640 medium with 10% endogenous EVs-free FBS for 24 h at 37 °C in a 5% CO_2_ incubator. Normoxic EVs and PBS, as controls, were added to each well, and the cells were further incubated for 12, 24, 48, or 72 h. The formazan solution dissolved with dimethyl sulfoxide (FUJIFILM, Osaka, Japan) was then added and incubated for 2 h at 37 °C. The colorimetric reaction products of reduced formazan were measured at 550 and 670 nm using a microplate reader (SH-1000Lab; Corona Electric Co., Ltd., Ibaraki, Japan). Subsequently, we investigated the effects of HA on EV uptake. Luc2-normoxic-EVs (10 μg/mL protein) incubated in 0.05% HA solution diluted in PBS for 1 h at 37 °C (EV-HA) or incubated in PBS (EV-PBS) were disseminated to UV2 cells (1.25 × 10^4^ cells) incubated in PBS with 1% FBS (EV free) under normoxic conditions after staining with Exo Sparkler (red), as mentioned above. UV2 cells were treated with EVs immersed in PBS as a control. UV2 endothelial cells were treated with a CD44 neutralizing antibody (R&D Systems) at 37 °C for 1 h. After 4 h of incubation with EV, UV2 cells were fixed in 1% PFA for 10 min and counterstained with DAPI (VECTOR Laboratories) for 15 min. Fluorescent images were taken with a BZ-x700 (KEYENCE), and the uptake rates of the extracellular vesicles were analyzed using ImageJ software. Statistical analysis was performed using Student’s *t*-test and Welch’s *t*-test.

## Data Availability

The original contributions presented in the study are included in the article/Supplementary Materials, further inquiries can be directed to the corresponding author/s.

## Supplementary Materials

The supporting information can be downloaded at https://www.mdpi.com/article/10.3390/ijms25179742/s1.
