# Supplementary material for: Role of CD44-Positive Extracellular Vesicles Derived from Highly Metastatic Mouse Mammary Carcinoma Cells in Pre-Metastatic Niche Formation
Source: Int J Mol Sci. 2024 Sep 9;25(17):9742. doi: 10.3390/ijms25179742 (PMC11395953; doi:10.3390/ijms25179742)
Supplement: Supplementary file 1 [file ijms-25-09742-s001.zip › ijms-3000161-supplementary.pdf]

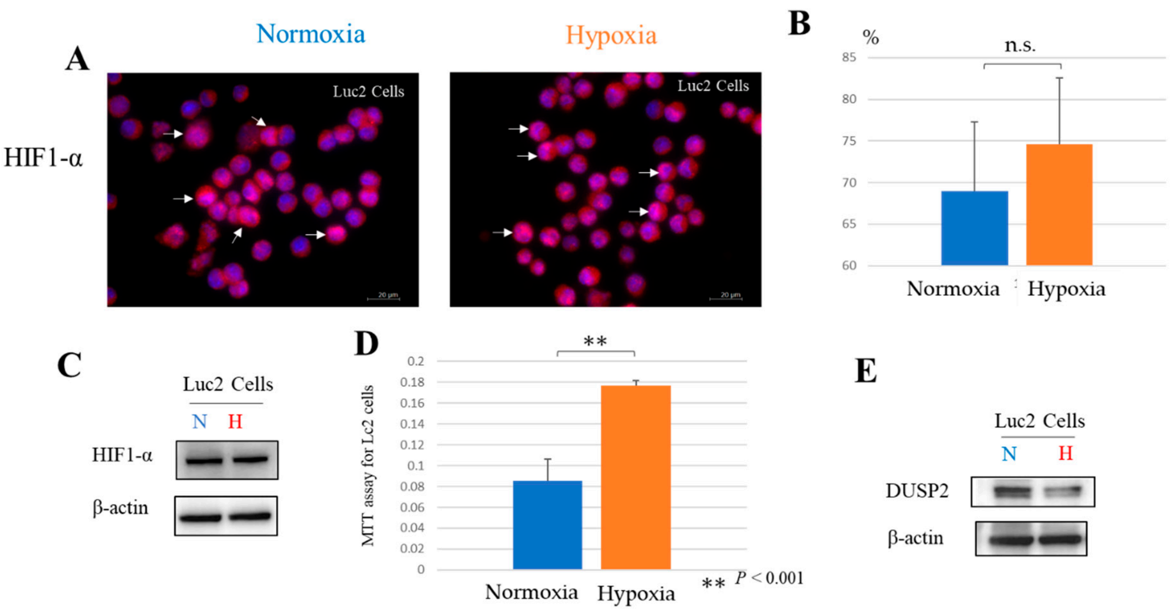

Supplemental Figure S1. EVs-donor Lc2 mouse mammary carcinoma cells response to hypoxic conditions. Fluorescence immunostaining (A) and Western blot analysis (C) were conducted on mouse mammary carcinoma cells (Luc2 cells) to assess the expression of hypoxia-induced factor 1 (HIF1-α) under normoxic and hypoxic conditions. The percentage graph of activated HIF1-α-positive cells (nucleus-positive cells in A) (B) and the MTT assay of cell proliferation rate in Luc2 cells under normoxic and hypoxic conditions (D) were also performed. Western bot analysis for another hypoxia-induced factor, the MAPK-specific phosphatase dual-specificity phosphatase-2 (DUSP2) (E). Scale bar = 20 µm.

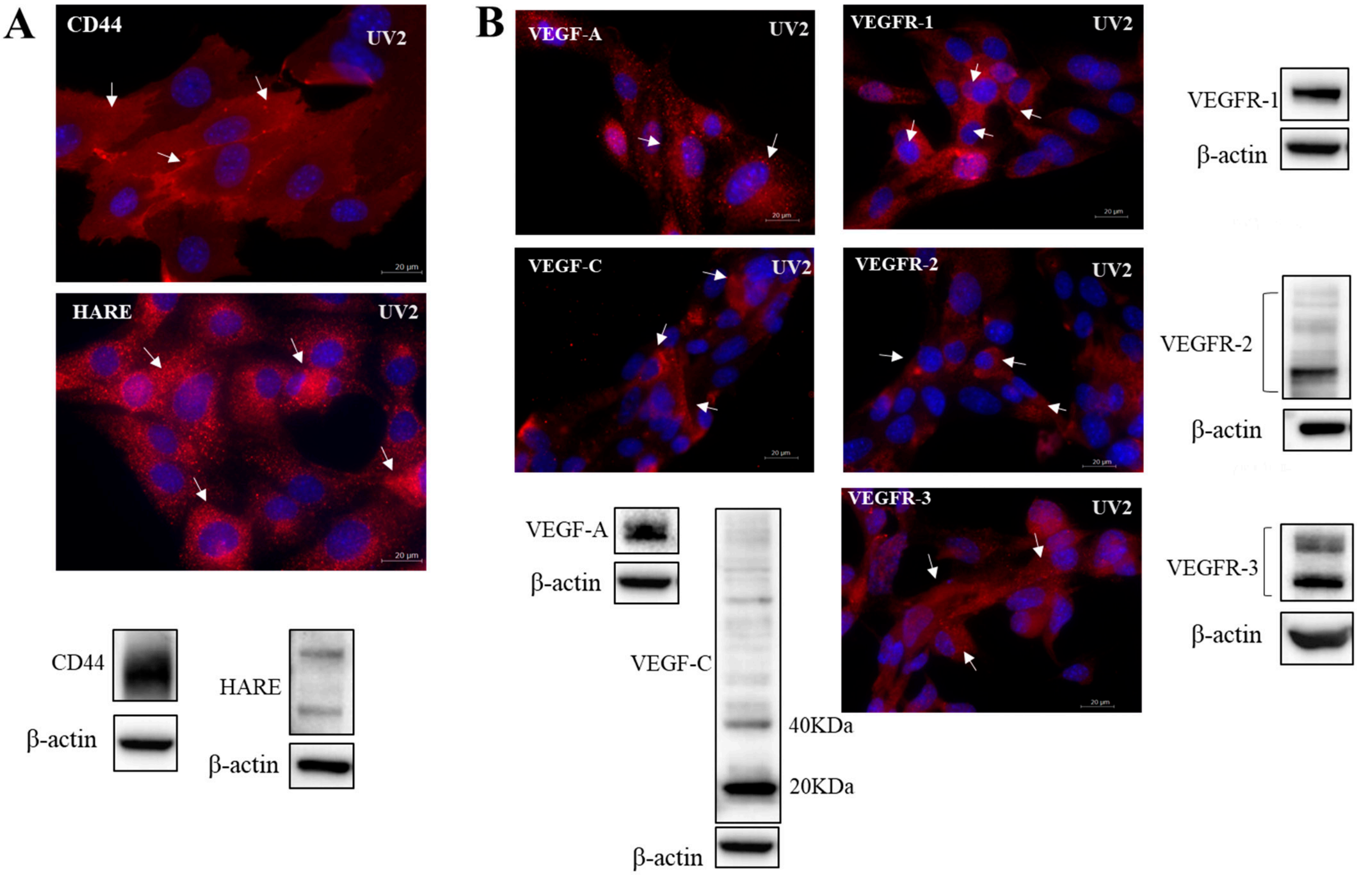

Supplemental Figure S2. UV2 endothelial cells (recipient cells of Luc2-EVs) express VEGF-A, VEGF-C, and their receptors. A: Immunofluorescent staining and western blot analysis of CD44 and hyaluronan receptor for endocytosis (HARE) under normoxic conditions. **B**, VEGF-A; VEGF-C; and their receptors VEGFR-1, -2, and -3 under normoxic conditions. The white arrows indicate the positive areas. Scale bar = 20 μm.
